# The Critical Role of Coefficients: Updating Allometric Normalisation Constants for Modern Ecology and Modelling

**DOI:** 10.1111/ele.70330

**Published:** 2026-02-06

**Authors:** Penelope S. A. Blyth, Thomas F. Johnson, Thomas Malpas, Hana Mayall, Alina Smith, Alain Danet, Eva Delmas, Christopher A. Griffiths, Benno I. Simmons, John Jackson, Ulrich Brose, Andrew P. Beckerman

**Affiliations:** ^1^ Ecology and Evolutionary Biology, School of Biosciences, University of Sheffield Sheffield UK; ^2^ Habitat Montréal Québec Canada; ^3^ Department of Aquatic Resources Institute of Marine Research, Swedish University of Agricultural Sciences Lysekil Sweden; ^4^ Centre for Ecology and Conservation, College of Life and Environmental Sciences University of Exeter Penryn UK; ^5^ Department of Conservation Biology Estación Biológica de Doñana (EBD‐CSIC) Seville Spain; ^6^ EcoNetLab, German Centre for Integrative Biodiversity Research (iDiv) Halle‐Jena‐Leipzig Leipzig Germany

**Keywords:** allometric coefficients, allometry, metabolism, normalisation constants, production

## Abstract

Allometry, the scaling of traits or biological rates with body mass, is central to a wide range of ecological research including dynamic food web modelling. There has been extensive focus on exponents (3/4 scaling laws), but little on the coefficients (normalisation constants). Coefficients that have been used since 2006 are derived from limited data and dated methodologies. Here, we compiled a data set of over 1000 genera with body mass spanning 10 orders of magnitude. We updated metabolism and production coefficients, deriving new genus and metabolic group levels estimates with phylogenetic hierarchical modelling providing robust inference. Our coefficients were mostly lower than those previously estimated, with increased uncertainty estimates. We used the Bioenergetic Food Web Model to evaluate their impact, finding increased biomass and species persistence but no change in stability. Our coefficients pave the way for future simulations that take advantage of subsets of genus and metabolic group data.

## Graphical abstract

Allometry is central to a wide range of ecological research including dynamic food web modelling. Here we updated the often neglected allometric coefficients for metabolism and production, deriving new genus and metabolic group levels estimates with phylogenetic hierarchical modelling providing robust inference. We show that using the updated coefficients influence the results of Bioenergetic Food Web Models, increasing total biomass and species persistence estimates.

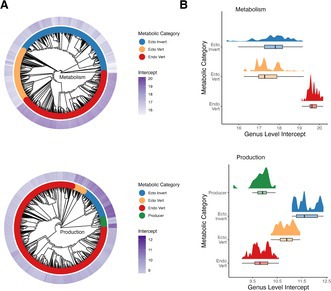

## 1 Introduction

Allometric relationships, which define how traits or rates vary with body size, are at the heart of the Metabolic Theory of Ecology (MTE) and a wide range of comparative analyses (Brown et al. 2004; Peters 1986). The functional form is typically *aM* ^*b*^, where *M* is body mass, *b* is an exponent defining the scaling and *a* is the allometric coefficient (a normalisation constant) most commonly used to differentiate among guilds or classes of species. Whilst the exponents have been extensively studied within and beyond the development of the MTE (Gillooly et al. 2001; Glazier 2005; Norin and Gamperl 2018; Savage et al. 2004; White et al. 2007; White and Kearney 2014; White and Marshall 2023), the allometric coefficients have received far less attention (Kaitaniemi 2008; Niklas and Hammond 2019).

Improving our knowledge of allometric relationships benefits many areas of ecology from trait‐based analyses to comparative life history studies (Font et al. 2019; Jackson et al. 2022) where accounting for phylogenetic distance is a crucial part of inference. Allometric coefficients are central to our understanding of how biological rates differ across taxonomic and metabolic groups, ecosystems and temperature ranges (Brose et al. 2019; Carter et al. 2023; Deutsch et al. 2020; Digel et al. 2011). They are also used to make inferences about rare or unmeasured species via imputation (Johnson et al. 2021; Riek and Bruggeman 2013).

Furthermore, they are a core component of recently developed mathematical models that are driving advances in multiple areas of biodiversity research. Perhaps the most widely used of these is the Bioenergetic Food Web (BEFW) model and its variant, the Allometric Trophic Network (ATN) model (Schneider et al. 2016) which simulate the biomass dynamics of tens to hundreds of species embedded in a network of consumer‐resource interactions (see Brose et al. 2006; Delmas et al. 2017; Lajaaiti et al. 2025; Schneider et al. 2016; Williams et al. 2007). These models have increased our understanding of stability (Brose et al. 2006; Domínguez‐García et al. 2019), species' persistence and diversity (Brose 2008; Stouffer and Bascompte 2010), robustness to primary and secondary extinctions (Binzer et al. 2011; Curtsdotter et al. 2011; Staniczenko et al. 2010), non‐trophic interactions (Kéfi et al. 2012), stressors and interaction among stressors (Binzer et al. 2012, 2016; Danet et al. 2025; Simmons et al. 2021) and ecosystem function (Delmas 2020; Miele et al. 2019; Rall et al. 2008; Schneider et al. 2016; Leroux and Schmitz 2025). Within these models, the coefficients help define key biological rates of populations. The BEFW and ATN models categorise individual species by metabolic groups—producers, ectothermic invertebrates, ectothermic vertebrates and endothermic vertebrates (Brown et al. 2004; Gillooly et al. 2001; Robinson et al. 1983)—and the allometric differentiation among the groups is defined by their allometric coefficients.

The current allometric coefficients specifically differentiate among metabolic groups, metabolism, production and foraging traits. These values were estimated and embedded in multiple implementations of the model from as early as 2006, when Brose et al. (2006) and then Williams et al. (2007) updated the Yodzis and Innes (1992) values based on emerging MTE data (Brose et al. 2006; Brown et al. 2004; Ernest et al. 2003; Gillooly et al. 2001; Williams et al. 2007). These coefficients therefore form rarely questioned assumptions, providing a foundation for numerous studies over the past two decades, influencing predictions about persistence, biomass and stability. Therefore, increases or decreases in any of these coefficients can potentially have marked impacts on the estimated rates of biomass loss (metabolism), production and transfer, thus influencing predictions of species persistence, community/trophic level/species biomass and the stability of ecological communities facing multiple stressors.

### 1.1 Improving Allometric Coefficients

There are two prevailing issues, and subsequent opportunities, in the coefficient estimates underpinning allometric relationships. First, sample sizes of traits and the number of taxa used for estimation were small. Whilst foraging rate estimates have been expanded to draw on 648 functional responses (Rall et al. 2012; Uiterwaal et al. 2022), the allometric coefficients for metabolism of invertebrates and biomass production of ectothermic vertebrates are, for example, still currently based on 20 and 9 data points, respectively (Brose et al. 2006; Ernest et al. 2003; Gillooly et al. 2001). Second, the statistical methods used to derive these historic values have not accounted for known sampling biases such as greater observations in species that are easier to measure or have received more research attention. Coefficients are therefore only representative of the biased statistical sample, not the statistical population or complete taxonomic groups. Further, by ignoring this bias, these approaches deliver conservative estimates of variation (uncertainty).

Both data volume and phylogenetic relatedness are important components in ensuring coefficients are representative of whole metabolic groups. Fortunately, in the nearly two decades since these parameters were introduced, the quantity of data available for coefficient estimation has increased dramatically, with substantial increases in the coverage across habitat types, taxonomic groupings, temperatures and body masses. Furthermore, the statistical tools available to estimate the coefficients have advanced, particularly those based on Bayesian hierarchical models and the implementation of phylogenetic covariance structures. By incorporating phylogeny we can account for the fact that more closely related species have similar traits via commonality of descent when considering the independence of data points. These approaches not only increase the robustness of the estimates, especially in the presence of biased taxonomic sampling, but also provide flexible estimates of uncertainty through their posterior distributions. This feature of Bayesian approaches ultimately offers unique opportunities to explore the sensitivity of predictions about stability, extinction dynamics and ecosystem processes to assumptions about the values of the parameters and develop models of communities aligned with specific subsets of species or functional groups.

Here, we introduce new estimates for the allometric coefficients for metabolism and the production of biomass (growth) across all metabolic classes. We do this by compiling a data set based on more than 20× the data used in the three key works that underpin the coefficients currently in use, published in 2004, 2006 and 2007 (Brown et al. 2004; Brose et al. 2006; Otto et al. 2007). We note that the 2006 coefficients remain the primary standard in 95% of publications using the bioenergetic model. We implement updated methods based on Bayesian Phylogenetic Least Squares (PGLS) modelling with specific incorporation of uncertainty. Allometric coefficients are then subsequently derived from the allometric intercepts of these new models.

We then explore the following questions: (1) Do higher volumes of data, representing substantially more taxa, alongside modern methods of PGLS, deliver estimates of allometric intercepts that are higher or lower than existing estimates? (2) Are estimates of variation (uncertainty) in the coefficients derived from these intercepts larger after accounting for phylogenetic non‐independence? (3) Is there variation in intercept estimates within and between metabolic categories? and finally (4) Do these new estimates change predictions about final biomass, species richness and stability when used in a bioenergetic food web model?

## 2 Material and Methods

We implemented a four‐step process to calculate and evaluate new allometric coefficients of metabolism and production rate. First, we executed a literature search to acquire data for each biological rate, temperature and the body mass of the taxonomic units. Second, we constructed a phylogeny for our compiled data. Third, we applied Bayesian phylogenetic least squares to estimate intercepts and their associated uncertainty. Fourth, we converted the intercept estimates into allometric coefficients for use in the bioenergetic food web modelling and assessed the impact of our new estimates on biomass dynamics, persistence and stability.

All statistical analyses were performed using R v.4.1.2 (R Core Team 2023) with packages including *tidyverse*, *revtools* for searching and acquisition of new data (Westgate 2019), *ape* and *rotl* for phylogeny construction and analysis (Michonneau et al. 2016; Paradis and Schliep 2019), and *brms* for Bayesian modelling statistics (Bürkner 2017). Food web simulations and dynamic models were run using Julia version 1.8.0 (Bezanson et al. 2017) and the *EcologicalNetworksDynamics.jl* package (Lajaaiti et al. 2025). We provide additional computational tool references for methods inline below.

### 2.1 Data Collection and Management

#### 2.1.1 Metabolic Rates

**FIGURE 1:**
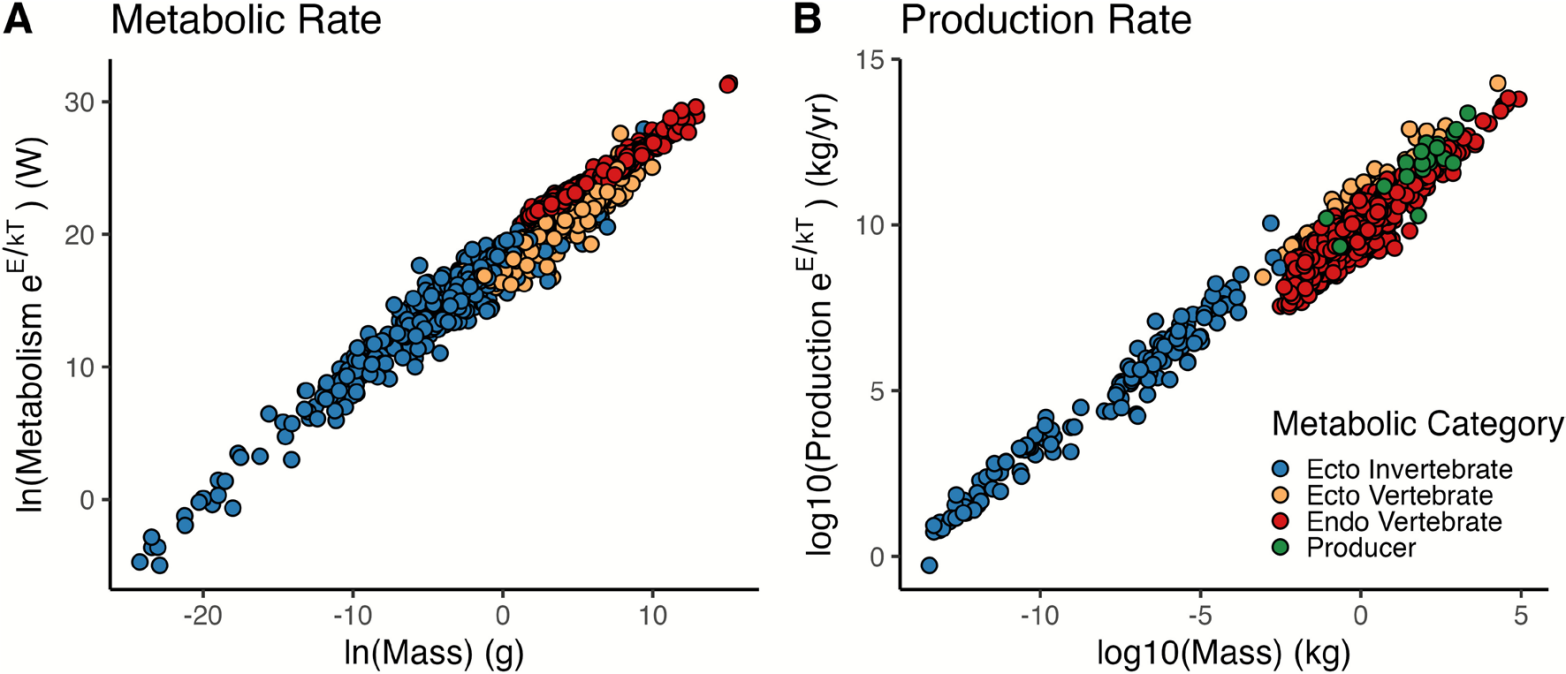
Temperature corrected biological rate variation according to body mass. (A) Metabolic rate in Watt; 1336 genera included. (B) Production rate in kilogram per year; 1005 genera included. Colours display the metabolic categories for all individuals separated into; Endothermic Vertebrate (red), Ectothermic Vertebrate (orange), Ectothermic Invertebrate (blue) and Producers (green).

We conducted a systematic literature search using the terms “(metabolic rate OR metabolism OR respiration rate) AND (body‐mass OR body‐size OR allometr*) AND (temperature OR warming OR cooling OR thermal)” on Web of Science, Scopus and the *revtools* R package (Westgate 2019) to generate a data set of metabolic rates. A total of 7632 measurements were collected for multicellular organisms ranging in body mass from 0.03 ng to 3672 kg and spanning 1336 genera: 552 ectothermic invertebrates, 259 ectothermic vertebrates and 525 endothermic vertebrates (Figure 1A) (Clarke et al. 2010; Ehnes et al. 2011; Gillooly et al. 2001; Makarieva et al. 2008; White et al. 2006).

Data includes individual body mass (*M*), temperature (*T*), taxonomic information and metabolic rate (*X*). Basal metabolic rate (BMR) was used for endotherms and standard metabolic rate (SMR) for ectotherms. *X* was recorded in Watts (*W*), for rates measured using the rate of oxygen consumption, a conversion factor of 20 J per 1 mL O_2_ was used (Makarieva et al. 2008). Temperature corrected rates (*Xe* ^*Ea/kT*^) were determined using Boltzmann's constant (*k*) and activation energy (*E*_*a*_) of 0.63 eV (Brown et al. 2004; Ernest et al. 2003; Gillooly et al. 2001). Body temperature was used for endotherms and environmental temperature for ectotherms. If no temperature was stated for an endotherm a default value was used; mammals 37°C, birds 39°C. (Ernest et al. 2003; Makarieva et al. 2008; White et al. 2006).

#### 2.1.2 Production Rate

Data for yearly production rates (*kg/yr*) of multicellular organisms was largely obtained from Hatton et al. (2019). This resulted in 2536 measurements of production (*R*) over 1005 genera spanning a magnitude of body masses from 0.035 ng to 84,639 kg: 19 producers, 142 ectothermic invertebrates, 46 ectothermic vertebrates and 798 endothermic vertebrates (Figure 1B).

Temperature corrected rates (*Re^Ea/kT^*) were determined as above. For non‐endotherm *R*, environmental temperature was taken from the original studies. If these data were not available, we obtained temperature values using the spatial coordinates of the study site. For terrestrial organisms, annual mean temperature was extracted using the study coordinates and Worldclim at a 2.5 m resolution (Fick and Hijmans 2017). For Marine organisms, sea temperature data was extracted from NOAA (NOAA 2015) by calculating the mean temperature of each species range polygon (IUCN 2022) at the midpoint of their depth range (www.fishbase.org; Froese and Pauly 2023).

### 2.2 Determining Taxonomy and Phylogeny

Where present, taxonomic information was acquired from the primary source. In cases where higher taxonomic levels were missing, we implemented an Open Tree of Life (OTL) approach using genera names and the OpenTree Synthetic Tree of Life version 13.4. We extracted taxonomy up to the Order level (OpenTreeOfLife et al. 2019).

Phylogenetic trees were constructed according to the genera subset specific to each rate and metabolic category (one tree for each metabolic category) with genera level tips using the OTL (OpenTreeOfLife et al. 2019) from the R package *rotl* (Michonneau et al. 2016). Branch lengths were assigned using the Grafen method and variance–covariance (VCV) matrices were created for each tree using the R package *ape* (Paradis and Schliep 2019) to include in the Bayesian PGLS analysis described in the next section.

### 2.3 Estimating Allometric Intercepts

#### 2.3.1 Statistical Models

New allometric intercepts were estimated for each metabolic category using three different Bayesian models, incorporating varying levels of phylogenetic information (Table S1). The base model (M1) is a Bayesian version of the original linear model (Brose et al. 2006) assessing how the temperature corrected rate varies with a fixed effect of genera mass, omitting all phylogenetic information. The taxonomy model (M2) builds on M1 by including taxonomic information as the nested term Order/Family and as a random effect. Finally, the Bayesian phylogenetic hierarchical model (PGLS; M3) builds on M1 and M2 by accounting for evolutionary relatedness between genera, via the variance–covariance matrix (VCV) determined from the phylogenetic tree. The combined use of Order and phylogenetic (covariance) random effects within M3 accounts for phylogenetic non‐independence among genera, allowing us to correct for evolutionary structure in our sample, that is, upweighting less sampled parts of the phylogeny (Paradis and Schliep 2019), increasing the alignment between the target population, in this case, all multicellular organisms, and our sample. Phylogenetic signal in the data was estimated in the model using Pagel's lambda (λ) (Freckleton et al. 2002; Symonds and Blomberg 2014).

These three models were fitted with the *brms* package in R (Bürkner 2017) using mean data for each genus and uninformed priors. They were separated by metabolic category; ectothermic invertebrates, ectothermic vertebrates and endothermic vertebrates, plus producers when examining production. We did not fix the exponent, so running separate models for each metabolic category allowed exponents to vary between groups (Table S3), consistent with the previous methodology (Brose et al. 2006; Ernest et al. 2003; Gillooly et al. 2001). A total of 21 models were fitted, 12 for production (3 models × 4 metabolic categories) and 9 for metabolism (3 models × 3 metabolic categories) each run until convergence ($\hat{R}\leq 1$) (see Table S1 for models, iteration numbers and units; Brose et al. 2006; Brown et al. 2004). Intercept values were extracted as the median intercept (C) for each metabolic category.

To allow for direct comparison with previous work, we fitted a frequentist ANCOVA model to the original data following Brose et al. (2006). This was used to verify that M1 produced comparable results to the original models. Intercepts for metabolism are measured in ln(W) and production in log10(kg/yr) (Brose et al. 2006).

#### 2.3.2 Models and Intercept Estimates

Q1: To assess the effects of increased data on intercept estimates, the ANCOVA and M1 were fitted using the data from 2006 and the new, more extensive dataset. The impact of including phylogenetic information on intercept estimates was tested by comparing the values produced using M1, M2 and M3.

Q2: The change in uncertainty around coefficient estimates from including phylogenetic non‐independence was compared by examining the 95% Confidence Interval (CI) from the original frequentist model (ANCOVA) and data, and the 95% Credible Interval (CRI) from our new data and PGLS model (M3). These metrics are from different statistical approaches; however, as the Bayesian models were run using uninformed priors and a large dataset, they produce CRIs that can be compared to CIs in practice (Albers et al. 2018; Bayarri and Berger 2004).

Q3: To assess variation in intercept estimates within and between metabolic categories we used the most appropriate model to the new data (M3) and generated intercept estimates at the metabolic group and genus levels. Genus‐level estimates were calculated.

By adding the predicted random effect level of that specific genera's Order and Phylogeny to their metabolic groups mean intercept. These estimates for both rates can be found in Appendix S2. Variation within metabolic categories is defined by the 95% CRI of genus level intercept values within that group. The differences between metabolic category level intercepts were assessed using the overlap of the 95% Credibility Intervals (CRI; posterior distributions) around each groups median intercept (Table 1).

**TABLE 1:** Comparison of allometric coefficients and the abbreviations used across rates and versions. This includes the original coefficient values (Yodzis and Innes 1992), currently accepted coefficients (Brose et al. 2006) and our new coefficients (Blyth) produced using the PGLS model and new data. Intercepts used to calculate the coefficient estimates for Brose and Blyth are noted along with their confidence (CI) or credible (CRI) intervals and statistical type (Frequentist or Bayesian). Estimates generated for Brose coefficients were calculated with data from Ernest et al. (2003) and Gillooly et al. (2001).

Category	Brose Intercept	Brose Freq CI	Blyth intercept	Blyth Bayes CRI	Brose coefficient	Blyth coefficient	Yodzis & Innes coefficient (kg^0.25^/yr)
Metabolism Intercepts ln(W)					a_x_	a_x_	a_T_
Ecto Invert	17.17	16.97 17.44	16.65	14.78 18.45	0.314	0.13	0.5
Ecto Vert	18.18	18.01 18.29	17.4	16.46 18.28	0.88	0.274	2.3
Endo Vert	19.5	19.37 19.78	19.53	18.93 20.13	3.22^a^	2.27	54.9
Production Intercepts log_10_(kg/yr)					a_r_	a_r_	a_r_
Producer	10.15	10.12 10.18	10.31	9.27 11.34	1	1	0.4
Ecto Invert	11.34	10.83 11.86	11.78	10.73 12.99	NA^b^	NA^b^	9.2
Ecto Vert	10.85	10.67 11.03	10.78	9.37 12.21	NA^b^	NA^b^	6.6
Endo Vert	10.29	10.25 10.33	9.83	9.16 10.51	NA^b^	NA^b^	34.3

^a^ Brose Endotherms a_x_ unpublished, calculated here using the original data and ANCOVA method comparable to that used in Brose et al. (2006).

^b^ Not published and unable to calculate from data used in Brose et al. (2006).

### 2.4 Effects of Allometric Coefficients on BEFW Outputs

**FIGURE 2:**
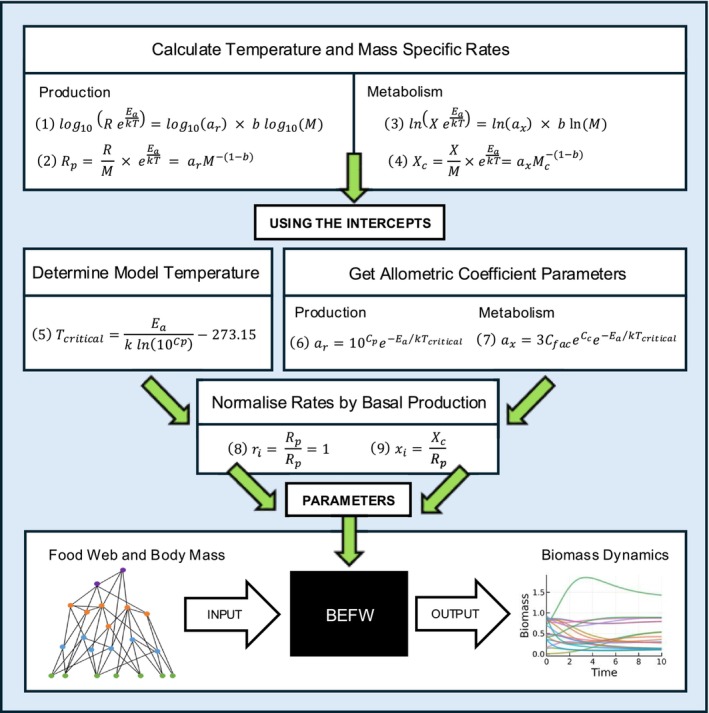
Intercept estimates to BEFW parameters. Top: Calculating mass and temperature corrected rates used to estimate allometric intercepts (*C*_*c or p*_) (Figure 1). Equations 1 and 3 show how production (*R*) and metabolic (*X*) rate for consumer (*c*) or producer (*p*) are temperature corrected using environmental (ectotherms and producers) or body temperatures (endotherms) (*T*), calculated with body mass (*M*), Boltzmann constant (*k*), activation energy (*E*_*a*_ = 0.63) (Brown et al. 2004), allometric exponent (*b*) and allometric coefficient (*a*_*r*_ and *a*_*x*_, respectively). Equations 2 and 4 are converting these rates to also be mass specific. Middle: Intercepts (*C*_*c or p*_) generated using equations 1 and 3, new data and PGLS model were used to calculate allometric coefficients in the form used in BEFW modelling (equations 6 and 7). To calculate these for the mass and temperature corrected metabolism (*x*_*i*_) a conversion factor (*C*_*fac*_ = 51.7) (Brose et al. 2006) is required, along with multiplying by 3 to convert from BMR to field metabolic rate (FMR) (Nagy 1987). Production and metabolic rates used in the BEFW model are normalised by the basal producer's production rate (*R*_*p*_) to infer a timescale (equations 8 and 9). One unit of time is defined as the inverse of the smallest primary producer's growth rate. This normalisation process assumes the critical temperature (*T*_*critical*_) at which models are run is the temperature at which the basal producer's mass and temperature corrected production rate is theoretically 1 (*r*_*i*_ = 1, equation 5, calculated by rearranging equation 6) (Delmas et al. 2017; Williams et al. 2007). For BEFW model simulation runs in subsequent sections, a *T*_*critical*_ of 34.6°C is used. To be consistent with the previous historical coefficient calculations, when re‐calculating the currently accepted coefficients with the original data and comparable ANCOVA model an *E*_*a*_ = 0.6 eV and *T*_*critical*_ = 24.883°C are used (Brose et al. 2006). Bottom: Simple schematic of a black box model of the BEFW.

Q4: Do these new coefficient estimates change predictions about final biomass, species persistence and stability when used in a bioenergetic model? To answer this, we needed to first translate the intercept estimates from the PGLS models into allometric coefficients which are parameters of the BEFW model. Figure 2 shows how metabolic group level intercepts estimated from our data and PGLS model are converted into the form of allometric coefficients of production (a_r_) and metabolism (a_x_) (equations 1–7) that are used in applications of the BEFW model. Exponents (b) for each rate and metabolic group can be found in Table S3.

We used the Julia implementation of the BEFW from Lajaaiti et al. (2025) to estimate the effects of the new coefficients on biomass dynamics, biodiversity dynamics and stability across several levels of connectance, species richness and predator–prey mass ratio (see Brose et al. 2006). We generated 800 food web structures using the niche model arising from 100 replicates each of a 3‐way factorial combination of two levels of connectance (*C* = 0.05 or 0.2), species richness (*SR* = 40 or 100) and predator‐prey body size ratio (*Z* = 10 or 100). *SR*, *C* and *Z* are key metrics driving food web structure and are the most consistently varied parameters in research on biomass dynamics, persistence and stability (Brose et al. 2006).

We ran the BEFW for each replicate food web using the currently published allometric coefficients and the median M3 coefficients for metabolism (*a*_*x*_ of ectothermic invertebrates and ectothermic vertebrates) and production (*a*_*r*_ of producers). We extracted final species richness, total biomass, population level stability (mean of the negative coefficient of variation (CV); Delmas et al. 2017, Brose et al. 2006) and community level stability (negative log average CV; Lajaaiti et al. 2025) from the last 100 time‐steps of each simulation, except community stability, which was extracted from the last 50. Because simulation studies can inflate sample sizes, reduce standard errors and deliver *p*‐values associated with small differences, we report η^2^ effect sizes from Type II sums of squares anova tables for the main effect of coefficient source (Brose vs. this paper) and for the interaction between source and the topological parameters *SR*, *Z* and *C*. We report these values for each of the four response variables. We used the *effectsize* package for R (Ben‐Shachar et al. 2020).

## 3 Results

We compiled data for metabolic and production rates from six published meta‐analyses, extracting data for 1336 and 1005 genera respectively, covering a wide range of body sizes, taxa and environment types. For example, the number of genera of invertebrates represented in the data for metabolism and production increased 39 and 13‐fold, respectively, when compared to the original dataset used by Brose et al. (2006). Rates were corrected for mass and temperature with organism masses ranging from 0.03 ng for parasitic protists to Bowhead whales ( *Balaena mysticetus* ) weighing over 84,000 kg (Figure 1) and temperatures between −11.4°C to 45°C.

Q1: Do higher volumes of data, representing dramatically more taxa, alongside modern methods of PGLS, deliver estimates of allometric intercepts that are higher or lower than existing estimates? Using the ANCOVA and M1 models, the addition of more data altered the allometric intercept values for all metabolic categories. Metabolic rate intercepts declined for ectothermic vertebrates and increased for ectothermic invertebrates and endotherms. The intercepts of production for all metabolic categories decreased except for producers, which increased (Figures S3 and S4).

**FIGURE 3:**
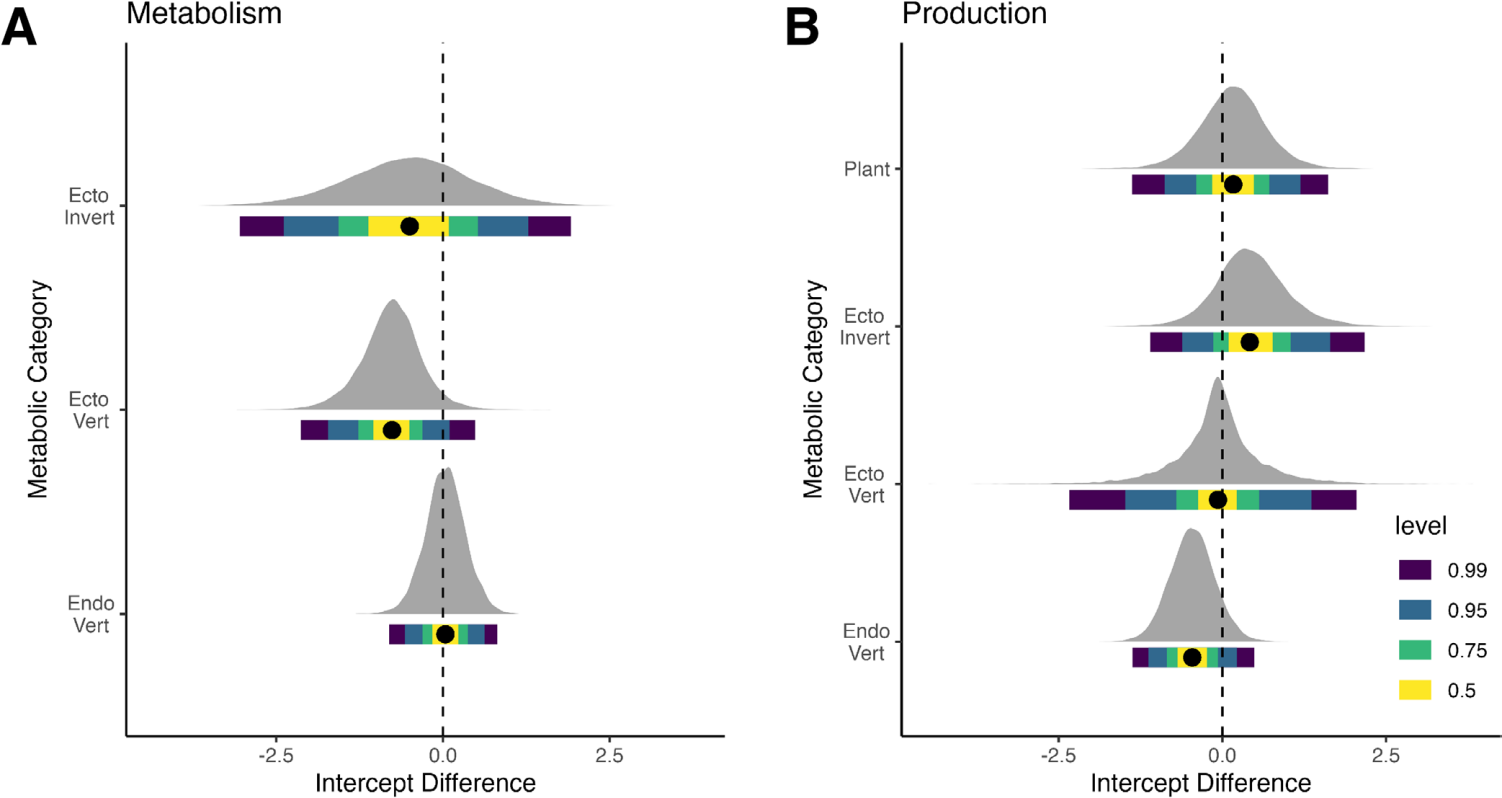
Comparison of Intercept value estimated from the PGLS model and new data to the currently accepted intercepts for (A) Metabolic rate and (B) Production rate. Posterior distribution around median intercept estimations (black dots) from PGLS (M3) shown for each metabolic category. No difference with previous intercept estimates for each metabolic category are shown by the dashed line with the x‐axis representing the difference in values of our new estimates relative to the previous estimates (Brose et al. 2006) for these graphs. Metabolism intercepts are calculated in ln(W), whilst production intercepts are (log_10_(kg/yr)). The credible intervals of our posterior distribution are represented by level, for example, 0.95 shows the 95% CRI.

The inclusion of phylogenetic information in the modelling approach (PGLS model combined with new data, M3) resulted in a further change in all intercepts compared to the original models (Figure 3A,B). Phylogenetic models of metabolic rate resulted in higher estimates for endotherms: 54.8% of the posterior distribution was above the current value. Ectothermic vertebrates and invertebrates had lower intercept values, with 92.6% and 71.4% of the posterior distribution below the current values. For production rates, invertebrate and producer estimates were higher, with 8.2% and 64.2% of the posterior distribution above the current values. The estimates for ectothermic vertebrates and endotherms were lower, with 57.5% and 91.1% of the posterior distribution below the current values, respectively.

Q2: Are estimates of variation (uncertainty) in the coefficients derived from these intercepts larger after accounting for phylogenetic non‐independence? Including any level of taxonomic information in model structure resulted in an increase in variation around median intercept estimates for all rates and metabolic categories (Figures S3 and S4). The largest variation resulted from the inclusion of phylogenetic relatedness in addition to taxonomic identity via the PGLS (M3).

Leave‐one‐out cross validation (LOO; Vehtari et al. 2017) showed that the addition of any level of taxonomic information improved predictive performance (Table S2). The best model for both rates varied between taxonomy (M2) and PGLS (M3) depending on the metabolic category examined. On average across metabolic categories, M3 had the best fit for production and M2 for metabolic rate.

As there was little difference in performance for M2 or M3 and both showed significant phylogenetic signal (metabolism λ: 0.58 to 0.87, production λ: 0.69 to 0.91, Table S3), we used the PGLS and the new data set to determine allometric intercepts and subsequent BEFW model coefficients. All models and convergence assessments are provided in Table S1 and Figures S1–S4. The increased variance can be further illustrated by the fact that all of the original 95% CI estimates (except for ectothermic vertebrate's metabolic rate) fell well within the 95% CRI produced by our new data and PGLS model (Table 1).

**FIGURE 4:**
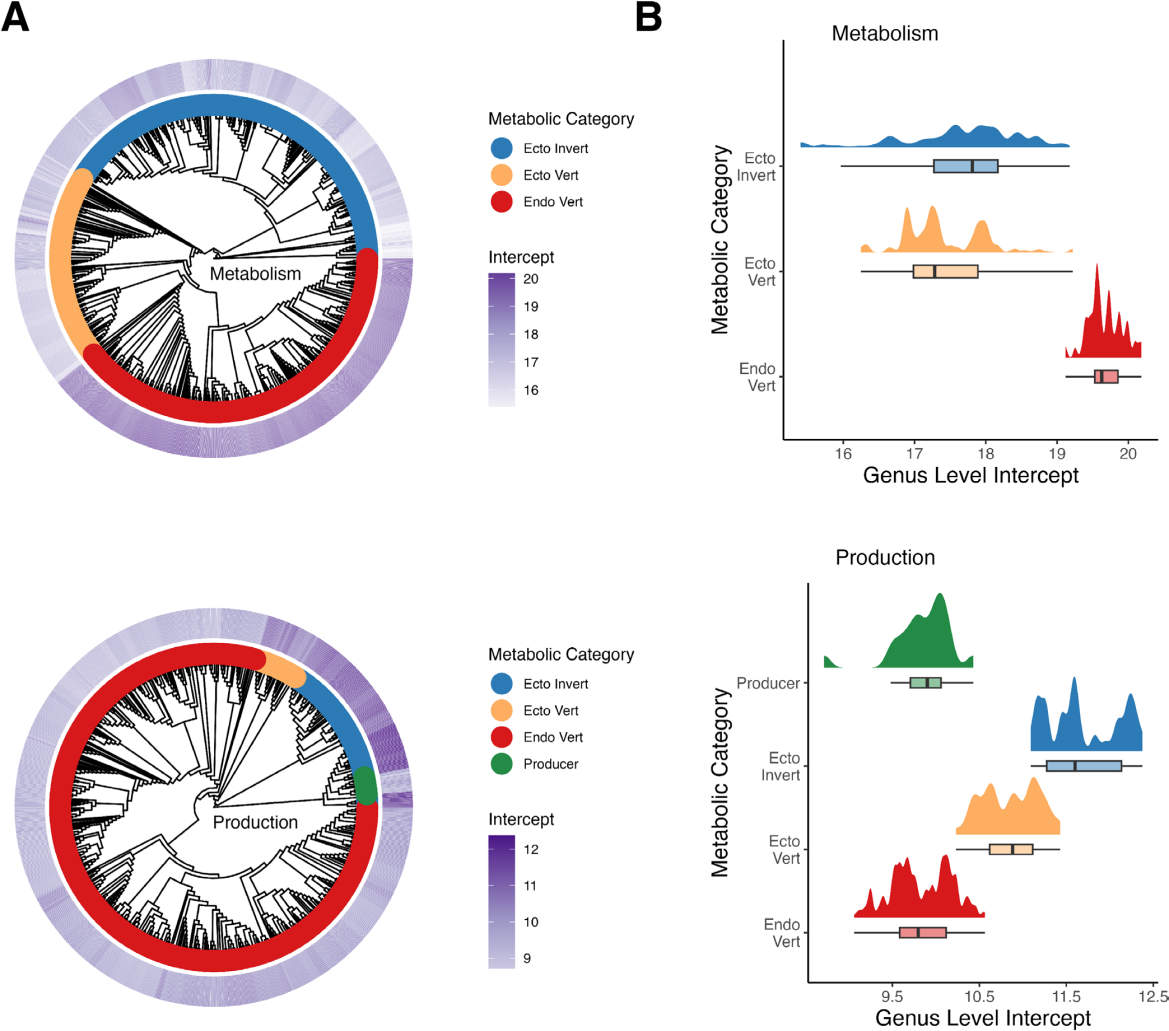
Variation within and between metabolic categories for intercept estimates of the allometric relationships for metabolic rate (top row) and production rate (bottom row). Intercepts for metabolism are measured in ln(W) and production in log_10_(kg/yr). (A) Cladograms displaying phylogenetic relatedness between genera used for Metabolic and production rate data. The inner band shows how the data is split between metabolic categories; endothermic vertebrates (red), ectothermic vertebrates (orange), invertebrates (blue) and producer (green). The outer band shows variation in genus level intercept estimate from phylogeny model (purple). (B) Cloud plot showing the distribution of genus level intercept values within each metabolic grouping.

Q3: Is there variation in intercept estimates within and between metabolic categories? Figure 4 highlights the distribution of intercept estimates from our PGLS model (M3) within and between metabolic groups in relation to phylogeny (Figure 4A) and each other (Figure 4B).

The largest variation of intercepts within a specific metabolic group belonged to ectothermic invertebrates' metabolism, with values from 19.18 ln(W) for a genus within Insecta to 15.4 ln(W) for a genus within Hydrozoa. This range was over 3 times larger than that found within endothermic vertebrates.

Whilst some metabolic groups are distinct from each other, the increase in intercept variation captured using the PGLS reduced the distinctions among metabolic groups overall. For production, endothermic vertebrates and ectothermic invertebrates' 95% CRI were distinct from each other but still overlapped with ectothermic vertebrates and producers. For metabolism, the endothermic vertebrates were the only metabolic group where the 95% CRI did not overlap at all with another group (Figure 4B, Table 1).

Q4: Do these new coefficient estimates change predictions about final biomass, species persistence and stability when used in a bioenergetic model?

**FIGURE 5:**
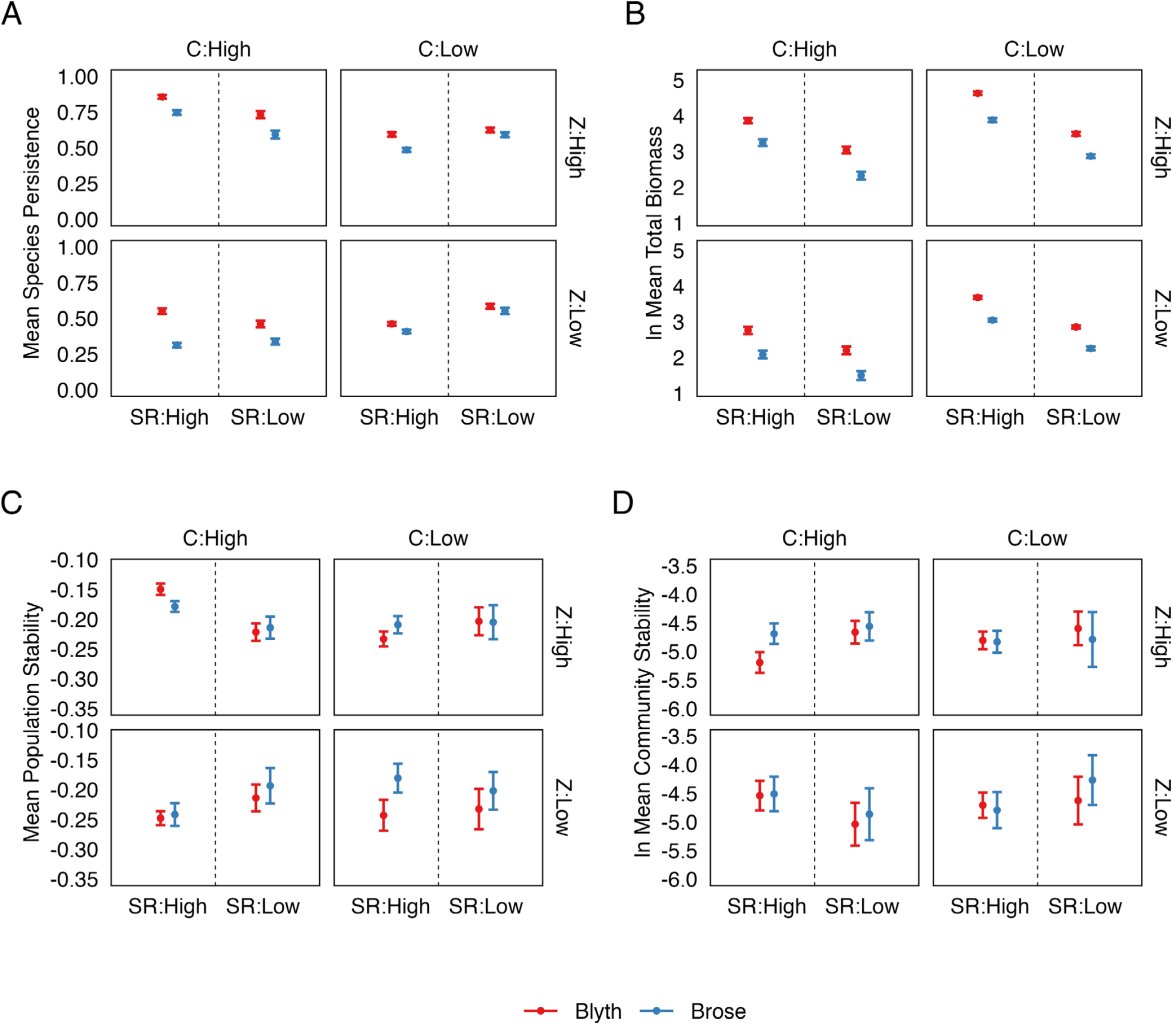
Effects of varying the allometric coefficients of metabolism of Invertebrates and Ectothermic Vertebrates on Bioenergetic Food Web (BEFW) model simulation outputs; (A) Mean Species Persistence, (B) Total Biomass, (C) Population level stability and (D) Community level stability.100 food webs were generated for each combination of High or Low values of connectance (*C* = 0.2 or 0.05), species richness (*SR* = 100 or 40) and predator prey body size ratio (*Z* = 100 or 10). The BEFW was run on each of these food webs, once using the currently accepted allometric coefficients (Brose, blue) and once with the new coefficients (Blyth, red) for a total of 1600 simulations. Error bars represent 95% confidence intervals.

This study produced new parameter values of *a*_*x*_ = 0.13 versus 0.314 for invertebrates, *a*_*x*_ = 0.274 versus 0.88 for ectothermic vertebrates and *a*_*x*_ = 2.27 versus 3.22 for endotherms (Table 1). When compared to BEFW outputs using Brose et al. (2006) values, these new coefficients altered outputs and predictions about the effects of species richness (diversity), connectance and the predator–prey mass ratio on biodiversity, biomass and population and community stability (Figure 5A–D, Table S4).

Interactions between structural (*SR*, *C* and *Z*) and allometric (Blyth or Brose) parameters on outputs resulted in either small or very small effect sizes (Table S4). The main effect of altering the coefficient resulted in a large effect size on species persistence (*F* = 282, *p* < 0.0001, *η* ^2^ = 0.151) and total biomass (*F* = 997, *p* < 0.0001, *η* ^2^ = 0.386), and a very small effect size on population (*F* = 6.97, *p* < 0.01, *η* ^2^ = 0.00437) and community level stability (*F* = 1.83, *p* = 0.177, *η* ^2^ = 0.00115).

## 4 Discussion

Here, we provide a comprehensive re‐estimation of allometric coefficients for metabolism (*X*) and biomass production (*R*). We present new coefficients with updated estimates of uncertainty derived from Bayesian hierarchical models accounting for phylogenetic relatedness. These parameters are vital for topics as wide ranging as differences in metabolic rates of fish at various organisational scales (Barneche et al. 2014; Norin and Gamperl 2018), the effects of temperature on carbon cycling in ecological networks (Yvon‐Durocher et al. 2010) and the impact of invasive species (Lurgi et al. 2014).

These coefficients often represent deep level assumptions about variation in biological rates among habitats, taxonomic, functional and metabolic groups in models of biodiversity dynamics. Ensuring their accuracy and estimating their precision (i.e., uncertainty) is therefore important to ensure that any inference gained is biologically realistic and precise. Estimating these intercepts with their uncertainty (here from posterior distributions) further offers opportunity for making more nuanced predictions of biomass, biodiversity and stability in a system and species‐specific manner.

### 4.1 Data Volume, Phylogeny and Diversity

The coefficients for metabolism and production have long been based on small sample sizes, low taxonomic and body‐size variation, and methods that omit phylogenetic information (Brose et al. 2006; Ernest et al. 2003; Gillooly et al. 2001). This combination of features provides limited insight into coefficient variation, restricting opportunities to evaluate the impact of metabolic and taxonomic variation on outputs from the models in which they were embedded.

It is important to consider the volume and taxonomic representation of data because it can increase the accuracy of mean estimates. Despite the increase in data here, further work is still needed to fill remaining gaps in order to improve future coefficient estimates. The proportion of mammal (61.6%) and bird (38.4%) genera within the Endothermic Vertebrate metabolic group is greatly different to the relative richness of these groups (Burgin et al. 2018; IOC, Gill et al. 2024). PGLS modelling, in part, helps account for the over‐ and under‐representation of certain groups, that is, phylogenetic non‐independence (Freckleton et al. 2002; Freckleton and Rees 2019; Johnson et al. 2024).

When there is phylogenetic structure in the residuals (a form of non‐independence in the sample data) estimates controlling for this typically lead to an increase in variation around the overall model parameters. However, by specifying the structure of the residuals (adding phylogenetic terms to the model), our prediction accuracy at the observation level increases. So, whilst a strong phylogenetic signal can increase uncertainty at the intercept, it can also improve predictability at the observation level (Johnson et al. 2021, 2024).

Introducing taxonomic information into our models increased the variance recorded around estimates (Figures S3 and S4). The 95% Credible Intervals from the Bayesian PGLS model represent a probability distribution that accounts for genus identity and metabolic classes. Capturing this variation is crucial for generating representative intercepts and the subsequent coefficients used to accurately predict changes in biomass, biodiversity and stability under climate change and multiple stressors.

### 4.2 Challenges and Opportunities Afforded by Bayesian PGLS

Current estimates of coefficients have been in use for two decades in the bioenergetic framework to run simulations on large food webs without the need for a fully resolved species list. This reduces data collection and computational time by running simpler models with fewer parameters and aims to avoid the over‐complication of models whilst still being able to recreate empirical dynamic patterns (Boit et al. 2012; Hudson and Reuman 2013). However, when such allometric relationships are derived from high volumes of data with taxonomic, metabolic and habitat representation, biodiversity modelling can advance from revealing general relationships between, for example, stability and complexity (Brose et al. 2006), to much more nuanced or focused questions about specific communities or habitats facing multiple threats.

#### 4.2.1 Opportunities

The increased range of sizes and taxonomic representation in our methodology allows for the estimation, with quantified uncertainty, of coefficients at the scale of the genus. These results can then be subset to genera that are present in a food web, representing real communities at the genus level. For example, the posterior distributions might allow longstanding questions about differences among marine, terrestrial and freshwater communities to be addressed by sampling from appropriate taxonomic and body size distributions (Brose et al. 2019; Digel et al. 2011; Valdovinos et al. 2023; Xiong et al. 2024), thus increasing precision in the use of the models for predicting impacts on biomass dynamics. Our new data and estimates facilitate this kind of inference, leveraging the Bayesian phylogenetic least squares estimation of posterior distributions. One might also use the data to simulate scenarios of spatial variation (Jordán et al. 2024; Galiana et al. 2021; Tekwa et al. 2022; Ryser et al. 2021) in biodiversity based on subsets of species embedding additional modelling of turnover and nestedness among species (e.g., β diversity) or temporal variation in species composition. Or as a foundation for also exploring evolutionary and life history related questions in dynamic, multi‐species systems where body size is the key trait (Loeuille and Loreau 2005; Luhring and DeLong 2020; Naisbit et al. 2012).

Beyond opportunities to leverage these data on dynamic food web models, such data will support continued work on the Metabolic Theory of Ecology (MTE) and an associated range of comparative trait analyses (e.g., functional traits (Messier et al. 2010) and behavioural traits (Nakagawa and Schielzeth 2012)) that explore the importance of variation, because a wider array of body sizes, taxonomy and ecosystems are represented (Brown et al. 2004; Isaac and Carbone 2010; Norin and Gamperl 2018; Peters 1986).

Furthermore, along with increasing the resolution of taxonomic information on which estimates are based (genus level), the PGLS model increases the potential future uses of biodiversity modelling across taxonomically unresolved data. Phylogenetic covariance in residual errors of the PGLS model on the data was recorded using Pagel's lambda (λ) (Freckleton et al. 2002; Pearse et al. 2025; Symonds and Blomberg 2014). Our models showed a strong phylogenetic signal opening up the possibility of imputation to infer coefficient estimates for the missing taxa, including those that are underreported (rare species), hard to measure or potentially newly discovered (Johnson et al. 2021; Riek and Bruggeman 2013). To assist with both of these points, our data and genus level intercepts are available in Appendix S2.

#### 4.2.2 Challenges

Despite these opportunities, we note that distinctions among metabolic groups have decreased. Using our PGLS approach, we observed a large amount of within‐group variation (Figure 4), resulting in overlap between some groups. Our data reveal a higher level of variability than has been assumed in the past. Endothermic vertebrates are often described as simply birds and mammals; however, some species of fish have been recorded to have either whole‐body endothermy such as Opah ( *Lampris guttatus* ) (Wegner et al. 2015) or regional endothermy (ability to maintain heat above ambient temperature in some but not all tissues) such as White Sharks ( *Carcharodon carcharias* ) and Atlantic Bluefin Tuna ( *Thunnus thynnus* ; Dickson and Graham 2004). As endothermy is associated with increased metabolic rates, inclusion of these ‘intermediate’ or regional endotherms into ectothermic vertebrates (which currently includes all other fishes) would falsely inflate their *a*_*x*_ value. As in the opportunities section above, the data actually allow a more nuanced and possibly accurate representation of biodiversity in specific communities.

### 4.3 Impacts of New Estimates on Biodiversity and Biomass Dynamics

The allometric relationships in the bioenergetic model define rates of biomass production (growth), loss (metabolism) and transfer among species (consumption), thus underpinning the dynamics that lead to patterns of persistence, biomass accumulation/loss and stability under different conditions (Brose et al. 2006; Delmas et al. 2017). They further allow researchers to resolve differences among metabolic groups and relate basic rates driving the model dynamics to groups of organisms/communities organised by body size. Increases/decreases in metabolic rate, for example, will lead to less/more biomass loss on average among species. The relationships deliver a different baseline of energy availability to a community defined by complex patterns of direct and indirect effects underpinned by production rates and foraging rates.

Most studies to date examining changes in population dynamics of a food web focus on the effect of functional response (Brose 2010; Kalinkat et al. 2013; Martinez et al. 2006), interaction strengths (Brose et al. 2019; Emmerson and Raffaelli 2004; Kartascheff et al. 2010; Tang et al. 2014), and trophic levels (Plitzko et al. 2012). Whilst some incorporate the allometry of metabolic rate (Brose et al. 2006; Heckmann et al. 2012; Kartascheff et al. 2010), there has to date been no opportunity to evaluate the effects of the coefficient values mapping through structural network characteristics (*SR*, *C* and *Z*) on bioenergetic output metrics (e.g., Blyth vs. Brose; Figure 5). This is critical to understand if we want to use the BEFW on as many different networks and ecosystem types as possible as no networks will be the same. Our study showed that altering allometric coefficients only had small or very small effects on the way structural metrics impacted species persistence and total biomass, and little to no impact on stability. We do note that estimates of both species persistence and total biomass do increase when the bioenergetic food web models were run using our new, lower *a*_*x*_. This may be due to lower *a*_*x*_ values reducing mass‐specific metabolic rate for each organism, decreasing consumer energy loss (e.g., from respiration), retaining more energy (biomass) in the system and reducing the pressure across trophic levels so fewer species are lost (Delmas 2020; Quévreux and Brose 2019). Yet overall, we conclude that whilst there are changes, they do not appear to alter the foundation for the last two decades worth of bioenergetic food web model studies and the qualitative insights (e.g., Gauzens et al. 2020; Miele et al. 2019; Schneider et al. 2016) are unlikely to be different. This is particularly important given the connected nature and sheer scope of works that use allometrically scaled metabolic rates (Schmitz and Leroux 2020; Schramski et al. 2015).

## 5 Conclusion

Our analysis has addressed a longstanding deficiency in data and modelling of key assumptions linking allometry of biological rates to the dynamics of biomass, biodiversity and stability. We highlight historic overestimation of intercepts, underestimation of variance and potential phylogenetic bias towards easy to study species. Our analysis also delivered novel intercepts for endothermic vertebrates that were previously not included at all for certain ecological models. Using our new allometric coefficients led to quantitative changes in biomass and persistence derived from bioenergetic food web models, but with limited impact on the qualitative role of structural parameters.

Perhaps the most important contribution of this work is the estimates of uncertainty which provide model coefficients that can define diversity in numerous ways—from metabolic groups to habitats to taxonomic levels as low as genus. We expect these new values and estimates of uncertainty will lead to a re‐assessment of previous work and opportunities for making biodiversity models more representative of the real‐world scenarios where climate change and multiple stressors are impacting ecological communities.

## Author Contributions

Initial concept was discussed by A.P.B., U.B. and P.S.A.B. P.S.A.B. conducted the analysis and wrote the first draft; advice on phylogenetic analysis was given by T.F.J. and C.A.G.; EcologicalNetworkDynamics.jl coding assistance by A.D., E.D. and T.M. and statistics advice by T.F.J., A.P.B. and B.I.S. Advice and support on data collection and additional drafts were given by T.F.J., T.M., H.M., A.S., A.D., E.D., C.A.G., B.I.S., J.J., U.B. and A.P.B.

## Funding

This work was supported by Natural Environment Research Council (NE/S001395/1 and NE/T003502/1).

## Conflicts of Interest

The authors declare no conflicts of interest.

## Supporting information


**Appendix S1:** ele70330‐sup‐0001‐AppendixS1.docx.

**Appendix S2:** ele70330‐sup‐0002‐AppendixS2.zip.

## Data Availability

The data and code to support this study's findings are available on Zenodo at https://doi.org/10.5281/zenodo.17170450.
