# Supplementary material for: The Critical Role of Coefficients: Updating Allometric Normalisation Constants for Modern Ecology and Modelling
Source: Ecol Lett. 2026 Feb 6;29(2):e70330. doi: 10.1111/ele.70330 (PMC12881220; doi:10.1111/ele.70330)
Supplement: Supplementary file 1 — Appendix S1: ele70330‐sup‐0001‐AppendixS1.docx. [file ELE-29-0-s002.docx]

**SUPPLEMENTAL 1**

**SUPPLEMENTAL TABLES**

**Table S1)** Model structure and variable units for each rate. Random effect is for random intercepts models; Taxonomy (M2) and PGLS (M3).

| **Rate/**  **Response** | **Model** | **Explanatory** | **Random effect** | **Statistical Type** | **Total Iterations**  **(Warm up \| Sampled)** |
| --- | --- | --- | --- | --- | --- |
| Metabolism  Ln Ie^E/kT^  (W) | ANCOVA | Ln mass  (g) | - | Frequentist | - |
|  | New Data (M1) | Ln mass  (g) | - | Bayesian | 6,000  (3,000 \| 3,000) |
|  | Taxonomy (M2) | Ln mass  (g) | Nested Order/Family | Bayesian | 6,000  (3,000 \| 3,000) |
|  | PGLS (M3) | Ln mass  (g) | Order  Phylogeny | Bayesian | 6000  (3000 \| 3000) |
| Production  Log_10_ Pe^E/kT^  (kg/yr) | ANCOVA | Log_10_ mass (kg) | - | Frequentist | - |
|  | New Data (M1) | Log_10_ mass (kg) | - | Bayesian | 6,000  (3,000 \| 3,000)* |
|  | Taxonomy (M2) | Log_10_ mass (kg) | Nested Order/Family | Bayesian | 6,000  (3,000 \| 3,000)* |
|  | PGLS (M3) | Log_10_ mass (kg) | Order  Phylogeny | Bayesian | 6,000  (3,000 \| 3,000)* |

*All models ran as stated for each metabolic category except for producer production rate. This ran for 10,000 iterations (5,000 warm up, 5,000 sampled).

**Table S2)** leave-one-out cross-validation (LOO) comparison for the output of each model structure on Metabolism and Production rates on each metabolic category (endo, ecto vert, invert, producer). Models Order from top (best fit) to bottom (worst fit). Models: New data (M1), Taxonomy (M2) and PGLS (M3).

| **LOO: Metabolic Rate** | | | |
| --- | --- | --- | --- |
| **Met Group** | **Model Order** | **elpd_diff** | **se_diff** |
| Endo | M2 | 0 | 0 |
|  | M3 | -10.1 | 8.2 |
|  | M1 | -131.0 | 14.3 |
| Ecto Vert | M2 | 0 | 0 |
|  | M3 | -9.5 | 5.5 |
|  | M1 | -64.3 | 11.4 |
| Invert | M2 | 0 | 0 |
|  | M3 | -5.3 | 11.8 |
|  | M1 | -160.9 | 21.6 |
| **LOO: Production rate** | | | |
| **Met Group** | **Model Order** | **elpd_diff** | **se_diff** |
| Endo | M3 | 0 | 0 |
|  | M2 | -16.8 | 10.5 |
|  | M1 | -472 | 33 |
| Ecto Vert | M2 | 0 | 0 |
|  | M3 | -5.3 | 3.3 |
|  | M1 | -17.7 | 4.7 |
| Invert | M3 | 0 | 0 |
|  | M2 | -7.7 | 2.9 |
|  | M1 | -20.1 | 5.8 |
| Producer | M3 | 0 | 0 |
|  | M1 | -5.9 | 2.6 |
|  | M2 | -6.1 | 1.3 |

**Table S3)** Comparison table of currently used allometric intercepts for metabolism and production rate compared to estimates produced using the PGLS model and new data. Phylogenetic signal shown by Pagel’s lambda (λ). Previous estimates were from Ernest *et al* (2003) and Gillooly *et al* (2001).

| Rate | Met cat | Current  Intercept | New  Intercept | CRI  Intercept | Slope | CRI  Slope | λ | #n  Genera |
| --- | --- | --- | --- | --- | --- | --- | --- | --- |
| Metabolism | Endo | 19.5 | 19.53 | 18.93  20.13 | 0.73 | 0.71  0.75 | 0.8 | 525 |
|  | Ecto Vert | 18.18 | 17.4 | 16.46  18.28 | 0.84 | 0.79  0.88 | 0.58 | 259 |
|  | Invert | 17.17 | 16.65 | 14.78  18.45 | 0.81 | 0.78  0.84 | 0.87 | 552 |
| Production | Endo | 10.29 | 9.83 | 9.16  10.51 | 0.75 | 0.72  0.78 | 0.91 | 798 |
|  | Ecto Vert | 10.85 | 10.78 | 9.37  12.21 | 0.9 | 0.82  0.98 | 0.82 | 46 |
|  | Invert | 11.34 | 11.78 | 10.73  12.99 | 0.87 | 0.8  0.93 | 0.73 | 142 |
|  | Producer | 10.15 | 10.31 | 9.27  11.34 | 0.79 | 0.55  1.01 | 0.69 | 19 |

**Table S4)** **Anova Table (Type II tests) and Effect Size (eta^2^)** from Anova models on how BEFW output varied with second level interactions of allometric coefficient (a_x_) with a topological factor Species Richness (SR), Connectance (C) or Predator-Prey mass ratio (Z).

| **BEFW Output** | **Interaction** | **F** | **p-value** | **Eta^2^** |
| --- | --- | --- | --- | --- |
| Species Persistence | a_x_:SR | 13.2 | 0.0002881*** | 8.24e-03 |
|  | a_x_:C | 59.5 | 2.107e-14*** | 0.04 |
|  | a_x_:Z | 1.33 | 0.250 | 8.33e-04 |
| Total Biomass | a_x_:SR | 0.0191 | 0.89 | 1.2e-05 |
|  | a_x_:C | 0.441 | 0.507 | 2.77e-04 |
|  | a_x_:Z | 0.355 | 0.552 | 2.23e-04 |
| Population Stability | a_x_:SR | 0.02 | 0.888 | 1.26e-05 |
|  | a_x_:C | 5.89 | 0.0153* | 3.69e-03 |
|  | a_x_:Z | 6.78 | 0.0093** | 4.25e-03 |
| Community Stability | a_x_:SR | 0.0013 | 0.971 | 8.44e-07 |
|  | a_x_:C | 1.386 | 0.239 | 8.71e-04 |
|  | a_x_:Z | 0.018 | 0.893 | 1.13e-05 |

**SUPPLEMENTAL FIGURES:**

**
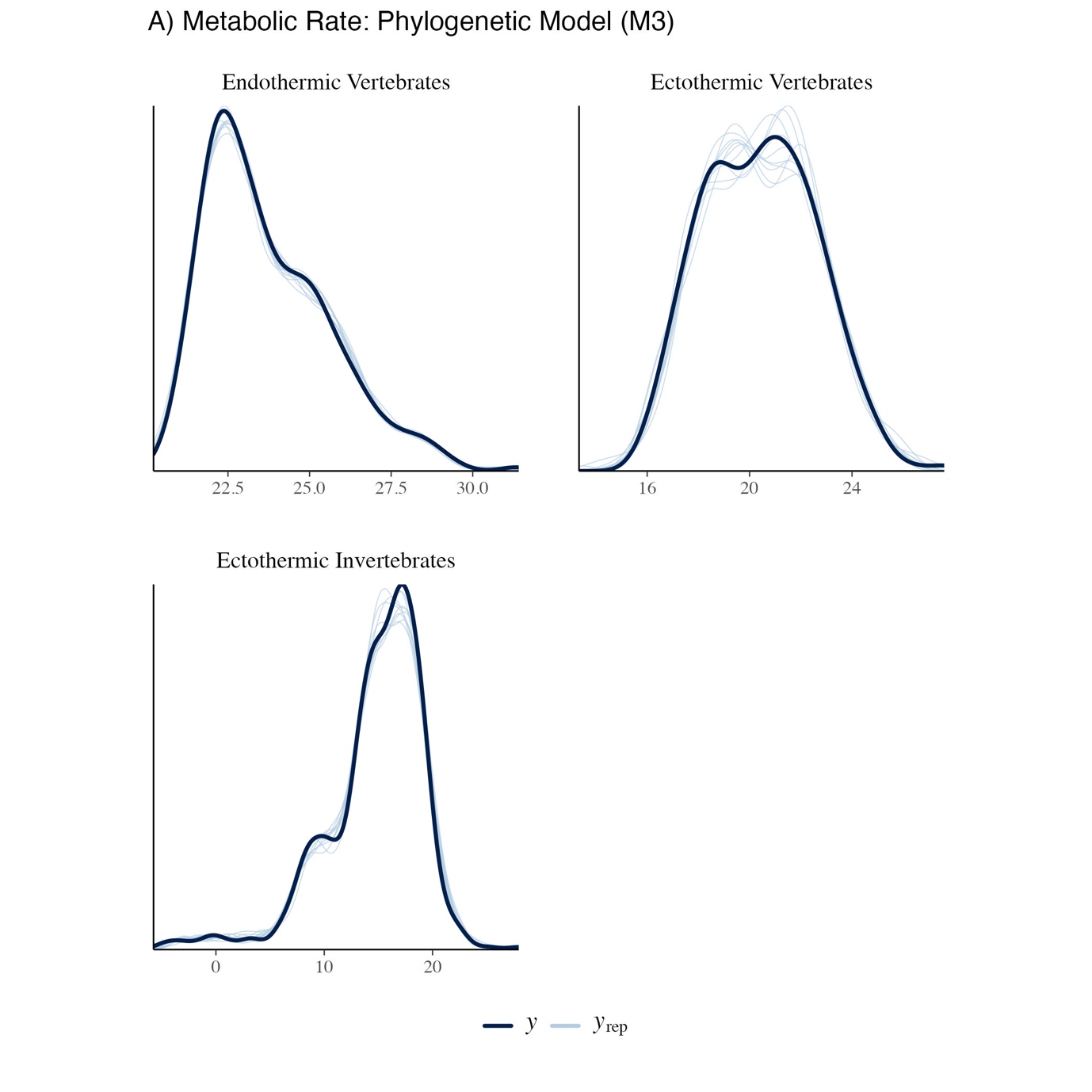
**


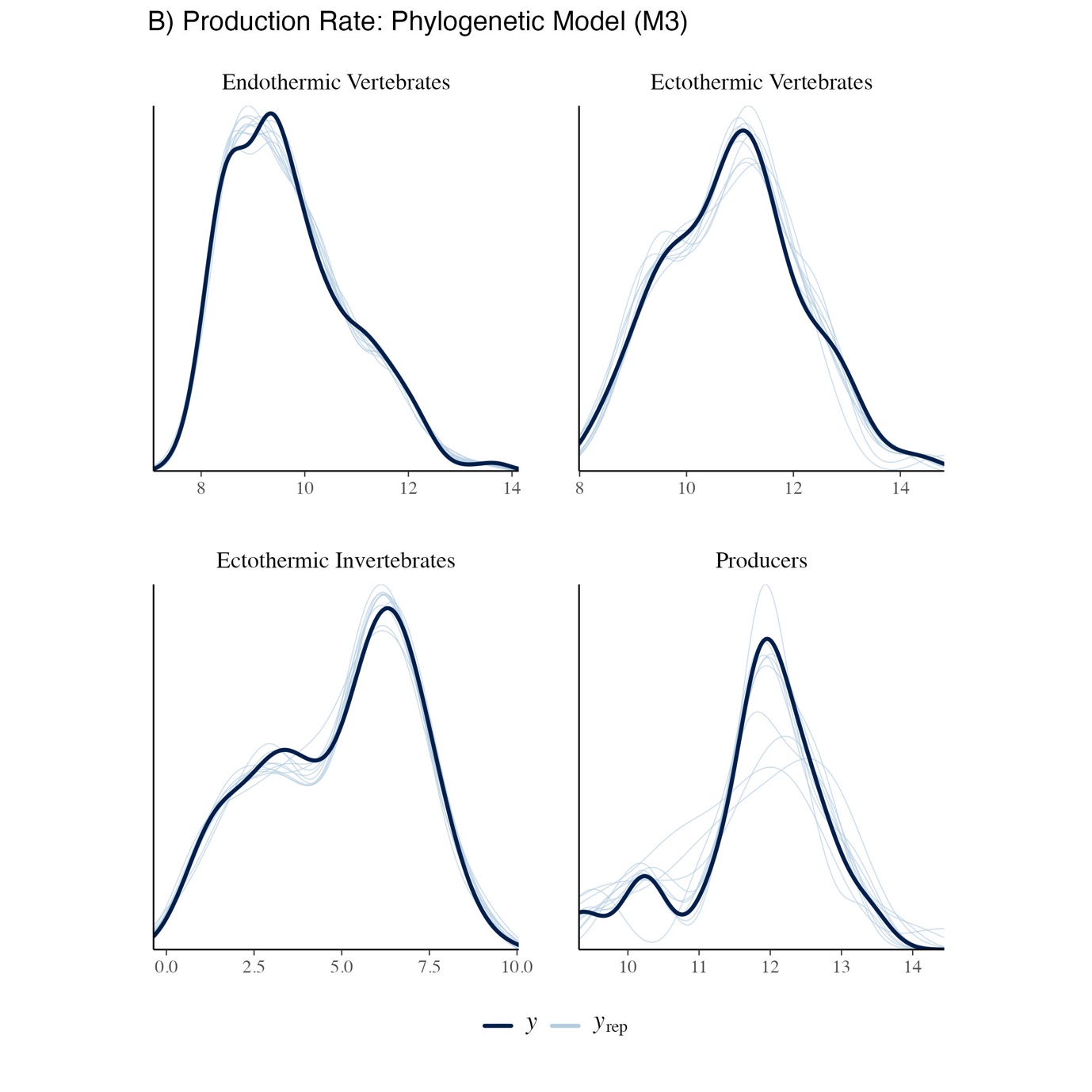


**Figure S1 – S2)** Posterior predictive checks for the phylogeny model (M3) on each rate A) Metabolism, B) Production.


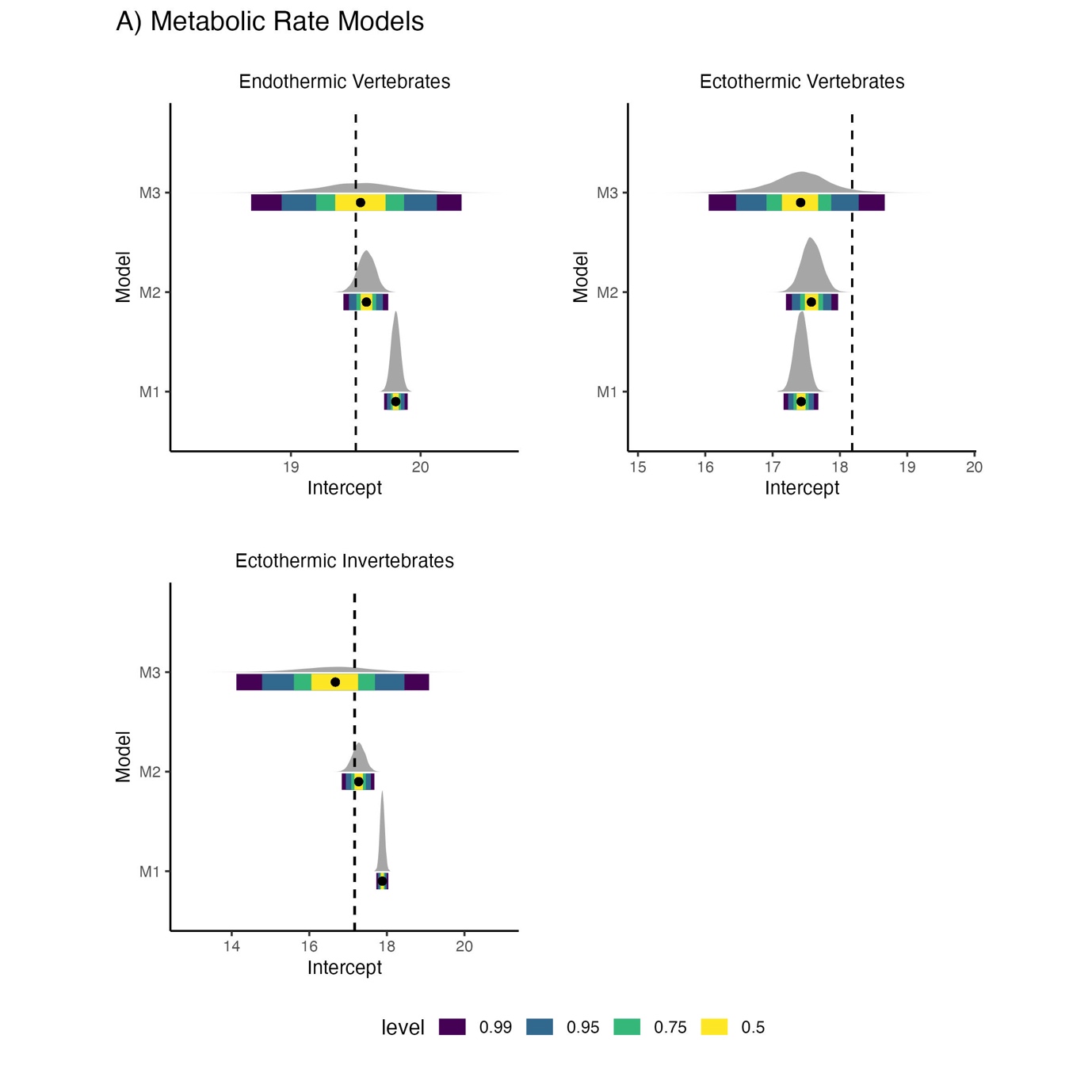


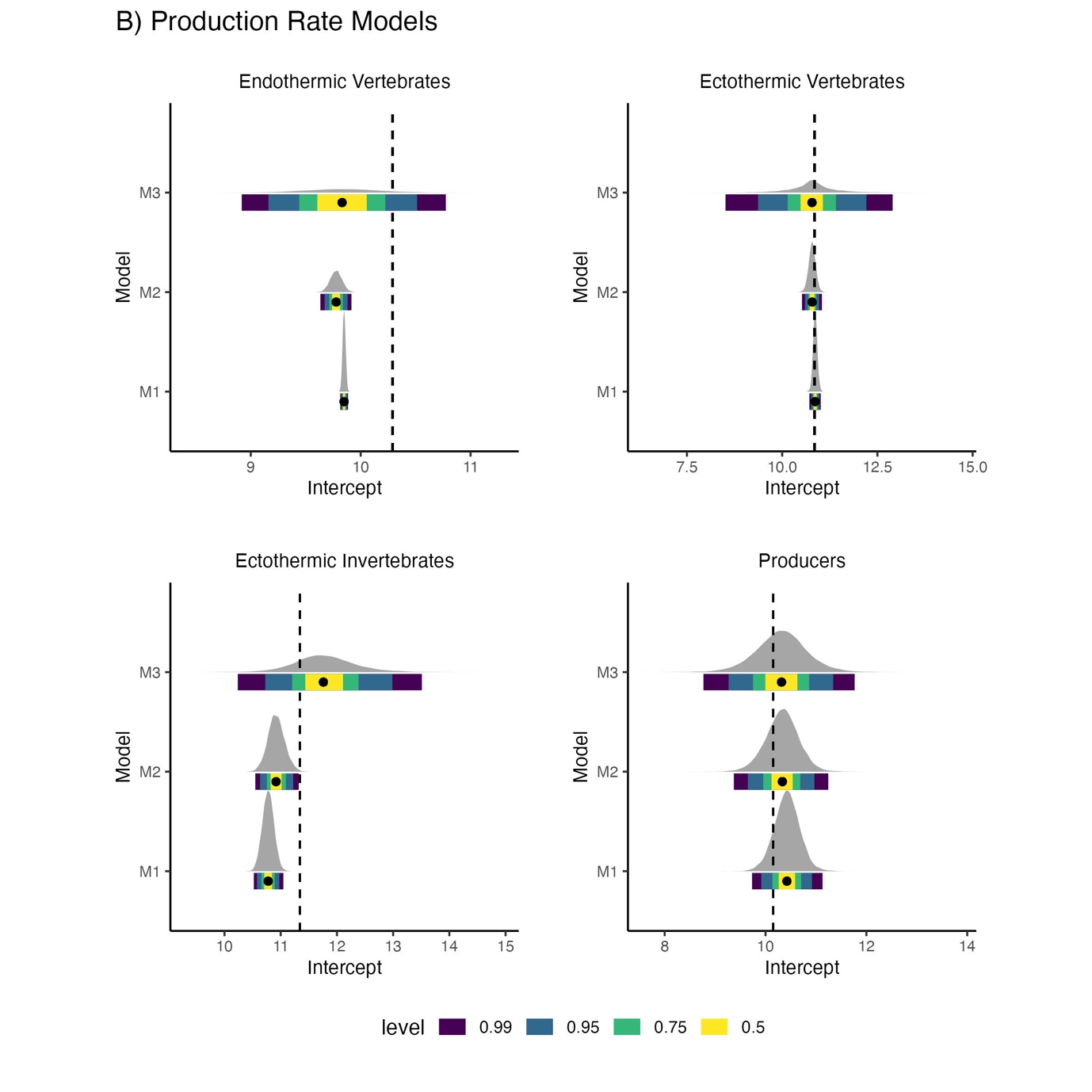


**Figure S3-S4)** Posterior distribution around median intercept estimations (black dot) from New Data (M1), Taxonomy (M2) and Phylogeny (M3) models on A) Metabolic rate, B) Production rate. Coloured bars show posterior distribution range. E,g, yellow 0.5 is 50% of the distribution in this band. Dashed line shows the currently used Brose intercept estimates (Brose *et al.* 2006).

**Supplemental References**

Brose, U., Williams, R.J. & Martinez, N.D. (2006). Allometric scaling enhances stability in complex food webs. *Ecol. Lett.*, 9, 1228–1236.

Ernest, M.S.K., Enquist, B.J., Brown, J.H., Charnov, E.L., Gillooly, J.F., Savage, V.M., *et al.* (2003). Thermodynamic and metabolic effects on the scaling of production and population energy use. *Ecol. Lett.*, 6, 990–995.

Gillooly, J.F., Brown, J.H., West, G.B., Savage, V.M. & Charnov, E.L. (2001). Effects of Size and Temperature on Metabolic Rate. *Science (80-. ).*, 293, 2248–2251.
