# Supplementary material for: The Critical Role of Coefficients: Updating Allometric Normalisation Constants for Modern Ecology and Modelling
Source: Ecol Lett. 2026 Feb 6;29(2):e70330. doi: 10.1111/ele.70330 (PMC12881220; doi:10.1111/ele.70330)
Supplement: Supplementary file 2 — Appendix S2: ele70330‐sup‐0002‐AppendixS2.zip. [file ELE-29-0-s001.zip › Supplemental_2_meta_data.docx]

**Supplemental 2: Meta-data** for production **(**genus_intercepts_production.csv) and metabolism (genus_intercepts_metaboism.csv)

Genus level estimates for temperature corrected production (P) and metabolism (X) allometric equations in the log form shown in equation 1 and 2. Equations were calculated using the PGLS model (M3) and input data available. Temperature correction is carried out on the rates prior to modelling using activation energy (E_a_) of 0.63eV, Boltzmann’s constant (k), and temperature in Kelvin (T).

$$\begin{aligned} {log}_{10} \left( P e^{\frac{E_{a}}{kT}} \right)=a \times{b log}_{10}\left( M \right) \#\left( 1 \right) \\ ln\left( X e^{\frac{E_{a}}{kT}} \right)=a \times b\ln\left( M \right) \#\left( 2 \right) \end{aligned}$$

| **Column** | **Information** |
| --- | --- |
| phylo | Genus name |
| gen_est_intercept | Genus level estimate of allometric intercept (a) |
| slope | Allometric exponent (b) calculated using M3 at metabolic category level |
| met_category | Metabolic Category;  Endothermic Vertebrate (endo_vert)  Ectothermic Vertebrate (ecto_vert)  Ectothermic Invertebrate (ecto_invert)  Producer (plant) |
